# Investigating Machine Learning Techniques for Predicting Risk of Asthma Exacerbations: A Systematic Review

**DOI:** 10.1007/s10916-024-02061-3

**Published:** 2024-05-13

**Authors:** Widana Kankanamge Darsha Jayamini, Farhaan Mirza, M. Asif Naeem, Amy Hai Yan Chan

**Affiliations:** 1 School of Engineering, Computer and Mathematical Sciences, Auckland University of Technology, Auckland, 1010 New Zealand; 2 Department of Software Engineering, Faculty of Computing and Technology, University of Kelaniya, Kelaniya, 11300 Sri Lanka; 3 Department of Data Science & Artificial Intelligence, National University of Computer and Emerging Sciences (NUCES), Islamabad, 44000 Pakistan; 4 School of Pharmacy, Faculty of Medical and Health Sciences, University of Auckland, Auckland, 1142 New Zealand

**Keywords:** Asthma, Risk of attack, Prediction, Machine learning

## Abstract

Asthma, a common chronic respiratory disease among children and adults, affects more than 200 million people worldwide and causes about 450,000 deaths each year. Machine learning is increasingly applied in healthcare to assist health practitioners in decision-making. In asthma management, machine learning excels in performing well-defined tasks, such as diagnosis, prediction, medication, and management. However, there remain uncertainties about how machine learning can be applied to predict asthma exacerbation. This study aimed to systematically review recent applications of machine learning techniques in predicting the risk of asthma attacks to assist asthma control and management. A total of 860 studies were initially identified from five databases. After the screening and full-text review, 20 studies were selected for inclusion in this review. The review considered recent studies published from January 2010 to February 2023. The 20 studies used machine learning techniques to support future asthma risk prediction by using various data sources such as clinical, medical, biological, and socio-demographic data sources, as well as environmental and meteorological data. While some studies considered prediction as a category, other studies predicted the probability of exacerbation. Only a group of studies applied prediction windows. The paper proposes a conceptual model to summarise how machine learning and available data sources can be leveraged to produce effective models for the early detection of asthma attacks. The review also generated a list of data sources that other researchers may use in similar work. Furthermore, we present opportunities for further research and the limitations of the preceding studies.

**Supplementary Information:**

The online version contains supplementary material available at 10.1007/s10916-024-02061-3.

## Introduction

Asthma, a common long-term lung condition, is caused by inflammation in the airways of the respiratory system. Asthma affected 262 million people in 2019, and it causes an average of 461,000 deaths each year [1]. Often beginning in childhood, asthma affects all ages [2]. A diagnosis of asthma considers clinical signs and symptoms, including wheezing, breathlessness, chest tightening, and coughing [3]. Asthma exacerbations could arise in patients due to multiple factors, including genetic, clinical, and environmental factors. For instance, if asthma patients are exposed to atmospheric changes such as dust, fumes, and pollen, their asthma can worsen, and it can lead to asthma attacks.

The reasons for asthma exacerbations are multifactorial, and worsening of asthma control may go unnoticed in patients if their symptoms are mild. However, asthma can worsen rapidly, leading to hospitalisation or death [4]. The National Review of Asthma Deaths (NRAD), conducted in the UK, found that 58% of patients with asthma who died were diagnosed as having mild or moderately severe asthma, with almost half having had no asthma review in the previous 12 months [5]. This highlights the need for innovative approaches to managing asthma and responding to attacks to ensure that care is delivered promptly. Another study [6] found that hospitalisation due to COVID-19 has increased among children (5-17 years) with poorly controlled asthma, as compared to children with well-controlled or without asthma. This highlights the increased risk that asthma patients face and emphasises the need for regular medical review, treatment, and proper management. This is important since asthma control can be affected by other factors, including environmental and meteorological conditions [7–9], workplace conditions, and severe adverse life events [10]. Therefore, early detection of asthma symptoms or exacerbations is essential to ensure rapid mitigation.

Even so, asthma prediction is challenging and complex due to its heterogeneous nature and the diverse multifactorial triggers unique to individuals [11, 12]. Patients with asthma are at a high risk of needing unscheduled medical care, which could be prolonged and costly to the health system. Any approach to predict asthma attacks will enhance asthma management and will reduce costs while increasing the quality of life. Whilst there has been a range of approaches to predicting the risk for asthma attacks that have been investigated in prior literature [13], the recent development of novel artificial intelligence techniques has seen a rapid rise in efforts to predict asthma exacerbations. However, the clinical relevance of these techniques and the potential to improve the accuracy and reliability of risk prediction is not yet known. There is a need to synthesise the available machine learning models that have been developed to a) identify the performance of these models in risk prediction and b) determine the range of clinical factors that have been included in these models. Understanding these factors is key if these models are to be used in routine practice for triaging patients based on their level of attack risk, or informing changes in their treatment based on their changing risk as part of clinical decision-making.

Machine learning (ML) techniques that have emerged to manage and treat various diseases are increasingly being applied, particularly in terms of risk prediction. ML is developed based on mathematics, logic, probability, neuroscience, and decision theory. Using these concepts, different algorithms are constructed that can store important information from data elements via continuous training sessions. Consequently, the models built upon these algorithms have the ability to identify patterns and generate outcomes from complex data without any human-generated explicit programming code [14]. These computer methodologies have been found to perform better than traditional statistical approaches that can facilitate personalised medicine by addressing individual patient characteristics while processing vast amounts of data [15, 16]. Previous studies have shown that ML models can support asthma monitoring, prediction, diagnosis, and control in children and adults. These techniques complement clinicians if expertise or resources are limited [17], preventing or mitigating tragic circumstances. This may be particularly true for exacerbation prediction as it will be difficult for health practitioners to consider a variety of factors, other than medical and clinical, in determining future attacks. Computer-based assistance can help predict impending attacks by considering a range of factors, and it will assist clinicians in making the appropriate decisions for the patient. The existence of a risk prediction model will be helpful for physicians to monitor disease control. It can also help patients as they will be made aware of any possible upcoming attacks.

**Fig. 1:**
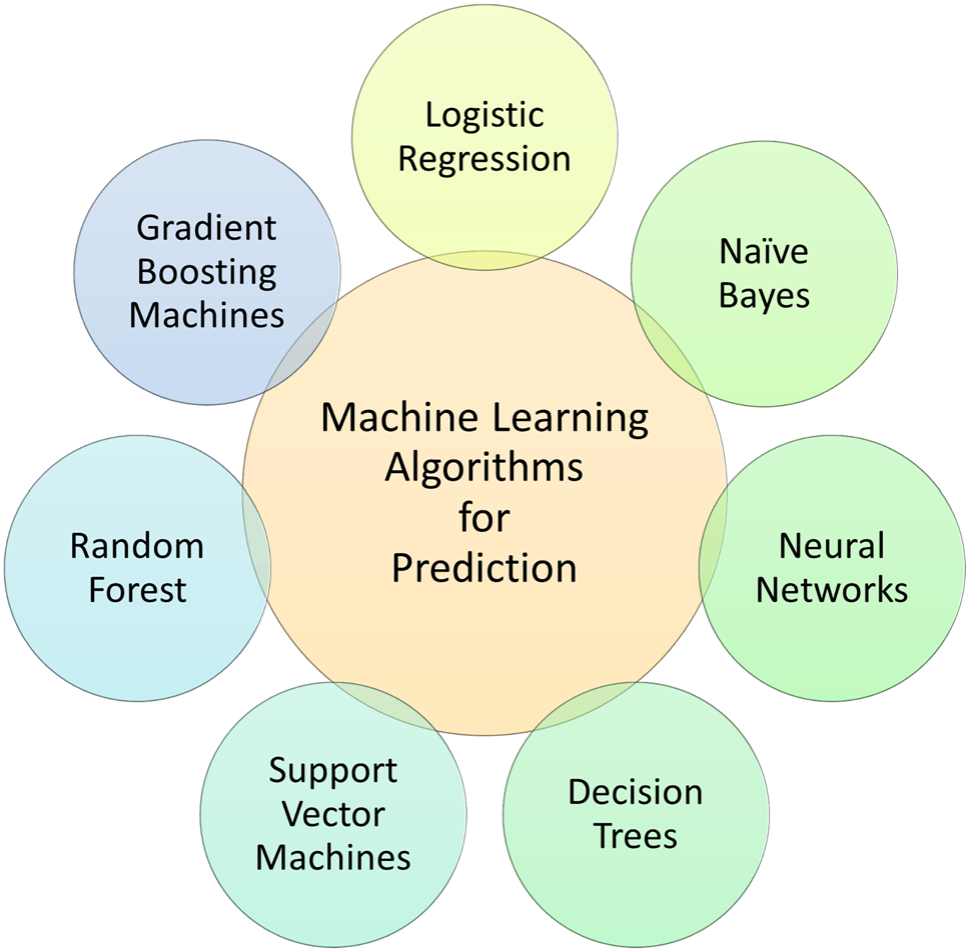
Commonly used machine learning algorithms for making a prediction

There are three categories of ML algorithms: supervised, unsupervised and reinforcement. Supervised algorithms are used to solve classification and regression problems. Typical applications of these algorithms are seen in spam filters, fraud detection systems, recommendation engines, image recognition systems, and so on. Figure 1 shows some of the commonly used ML algorithms for making predictions.

This systematic review explores the application of ML techniques to predict the risk of asthma attacks. The review question was "How do machine learning techniques perform in predicting asthma attacks?". Specifically, we identify the ML techniques used, categorise the predictive models based on the outcome, and identify the suitability of ML approaches as they relate to the scenarios we analysed. We also identify the best-performing ML algorithms and provide prospective research directions.

The subsequent sections of this paper are organised as follows. Section “Methodology” explains the methodology employed in this investigation, while “Results” section articulates the findings derived from the study. Section “Discussion” provides an in-depth discussion of these findings. Section “Conclusion” summarises the study and suggests potential avenues for future research.

## Methodology

This review adopted the Preferred Reporting Items for Systematic Reviews and Meta-Analyses (PRISMA) methodology [18]. The protocol has been registered with the PROSPERO International Prospective Register of Systematic Reviews (PROSPERO CRD42023402178).

### Inclusion Criteria

This study included primary studies of ML-based solutions for asthma in adults and children published in English from January 2010 to February 2023. No limitations were placed on the study design, setting, or minimum follow-up, but studies were limited to the English language due to the language capabilities of the research team.

### Exclusion Criteria

Studies focusing on the diagnosis/prediction of asthma itself (as a condition), rather than asthma exacerbations, were excluded from the current study. Any studies that have followed a non-machine learning approach were excluded. Additionally, studies focusing on predicting asthma symptoms/control/severity level, peak expiratory flow rate (PEFR) and emergency department (ED) visits due to other diseases were eliminated from the study pool. Further, we excluded research work published as reviews, systematic reviews, editorials, letters, comments to the editor, book chapters, abstracts, conceptual papers, opinions, unavailable sources, protocols, commentary, and unpublished full-text documents.

### Data Sources and Search Strategy

We searched the databases Medline (via PubMed), Cochrane (via Wiley), Embase, IEEE Xplore, and Google Scholar using the search terms Asthma AND (attack* OR exacerbat* OR control* OR symptom*) AND (detect* OR predict* OR diagnos* OR manag*) AND ("artificial intelligence" OR AI OR "machine learning" OR "deep learning" OR "neural network" OR computer-based OR "computer based" OR computer-assisted OR "computer assisted" OR "computer technology" OR technology).

### Selection Process

**Fig. 2:**
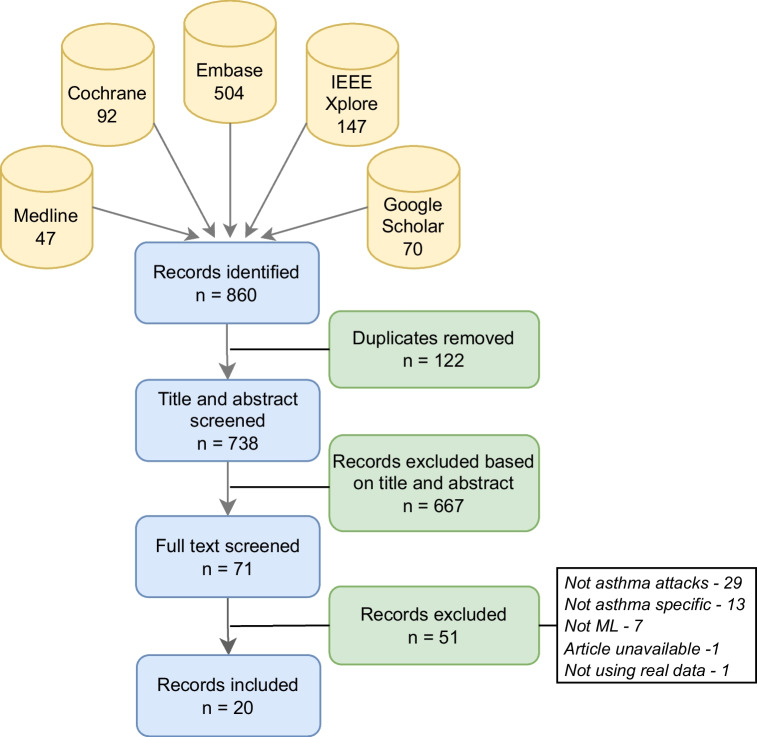
Study inclusion process via PRISMA

The database search identified 860 eligible records. We used the tool RAYYAN for the study selection process [19]. After removing 122 duplicates, the titles and the abstracts of the remaining studies were screened. At the end of this step, we eliminated 667 records, and the rest of the studies were sent through full-text screening, which resulted in the selection of 20 studies to be included in our review. These were blindly reviewed by two different researchers. This study selection process is illustrated in Fig. 2.

### Data Extraction

A data extraction table was created to record the specific data extracted from the selected studies. The design of the table structure was informed by previous research [20, 21]. We extracted the year, country, title, aim, data source/s, sample size, research techniques, findings, evaluation metrics, model performance, and the limitations and strengths of each study.

### Risk of Bias Assessment

A risk of bias assessment of the selected studies was conducted using the Critical Appraisal Skills Programme (CASP) for Clinical Prediction Rule [22]. The CASP checklist was completed for each study independently by two authors. Any disagreements were resolved by consensus discussion. An additional file shows the quality of each study (see Supplementary file S1).

## Results

### Characteristics of Included Studies

**Fig. 3:**
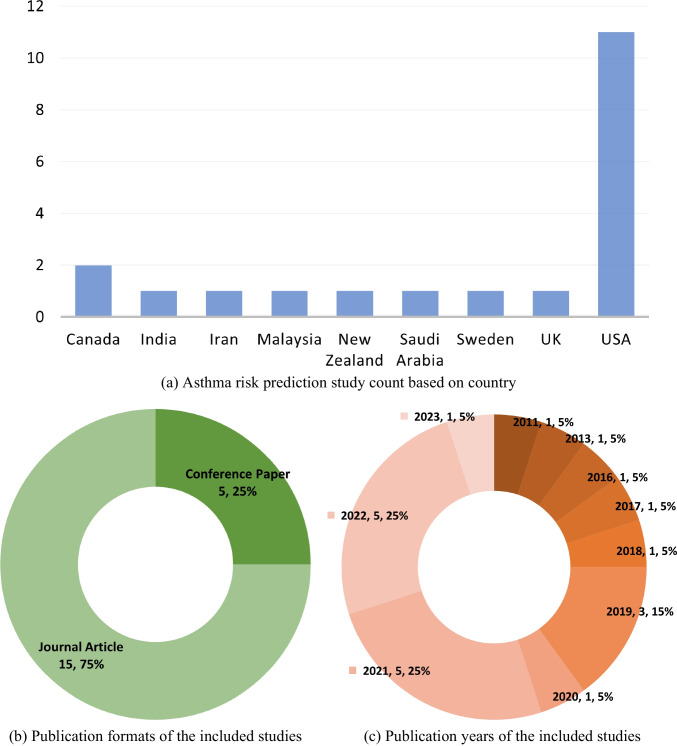
Characteristics of the included studies

Characteristics of the 20 studies included in this review are elaborated in Fig. 3. The included studies were conducted in a range of different countries, represented in Fig. 3a. (A study [23] that used an international data set from multiple countries is not included in the figure.) Importantly, as can be seen from Fig. 3a, the USA is the main country of origin for many of these studies predicting asthma attack risk using ML techniques. As shown in Fig. 3b, 75% of the studies were published as journal articles and the rest as conference papers. Figure 3c shows the distribution of the studies by year of publication. The majority of the studies have been published after 2020. The data sets employed in these studies have different sample sizes according to the number of participants or records (data instances). These details are presented in Table 1, showing the absolute values and percentages. Table 1 highlights the distribution of the two classes of the target variable: asthma attacks, both absent and present, as well as the portions of data used for training and testing the prediction models.

In this review, we identify the data sources that the previous studies incorporated to predict the risk of asthma attacks. Table 2 shows the different data domains including biological, clinical, environmental and meteorological, hospital and medical, and socio-demographic that have been used to develop the asthma risk prediction models. Clinical data may include asthma symptoms, PEFR, and inhalations while prescribed medications and treatments come under medical data. Hospital data consists of hospital admissions, previous attacks, comorbidities, ED visits, etc.

**Table 1:** Characteristics of datasets used in previous studies

**Study**	**Data sample size (total)**				**Training data**	**Testing data**
	**#Patients (P)**	**#Records (R)**	**Asthma attack absent**	**Asthma attack present**		
[24]	266	132,972	179 P (67.3%)	87 P (32.7%)	165 P (62%)	101 P (38%)
[25]		3,470	3,350 R (96.5%)	120 R (3.5%)	3,473 R (70%)	997 R (30%)
[26]		2435	2,287 R (93.9%)	148 R (6.1%)	1,452 R (70%)	983 R (30%)
[27]	29,396				17,638 P (60%)	11,758 P (40%)
[28]	298		234 P (78.5%)	64 P (21.5%)	184 P (60%)	60 P (20%)
[23]		728,535	727,959 R (99.9%)	576 R (0.1%)	582,828 R (80%)	145,707 R (20%)
[29]	5,982	17,907	12,862 R (71.8%)	5,045 R (28.2%)	4,008 P (67%)	1,974 P (33%)
[30]	3,057				2,447 P (80%)	610 P (20%)
[31]		334,564	322,420 R (96.4%)	12,144 R (3.6%)	315,308 R (94%)	19,256 R (6%)
[32]	3,678		2,855 P (77.6%)	823 P (22.4%)	2,942 P (80%)	756 P (20%)
[33]	109,488				98,823 P (90%)	10,665 P (10%)
[34]	21	655			524 R (80%)	131 R (20%)
[35]	240		106 P (45.4%)	134 P (54.6%)	216 P (90%)	24 P (10%)
[36]	10					
[37]		12,000			9,000 R (75%)	3,000 R (25%)
[38]		29,392	24,435 R (83%)	4957 R (17%)	23,514 R (80%)	5,878 R (20%)
[39]	581		404 P (69.5%)	177 P (30.5%)	417 P (72%)	164 P (28%)
[40]	60,302				48,242 P (80%)	12,060 P (20%)
[41]	178	3,970			3,000 R (75%)	970 R (25%)
[16]	31,433		29,171 P (92.8%)	2,262 P (7.2%)	25,146 P (80%)	6,287 P (20%)

**Table 2:** Data sources used in previous studies

**Data source**	**Previous studies**
Biological	[39]
Clinical	[16, 23–26, 28, 31, 32, 34, 37, 39, 40]
Environmental and meteorological	[34, 36, 38, 39, 41]
Hospital and medical	[16, 27, 28, 30, 31, 33, 35, 39–41]
Socio-demographic	[16, 27–29, 31–33, 36, 38–40]

### ML Models

**Fig. 4:**
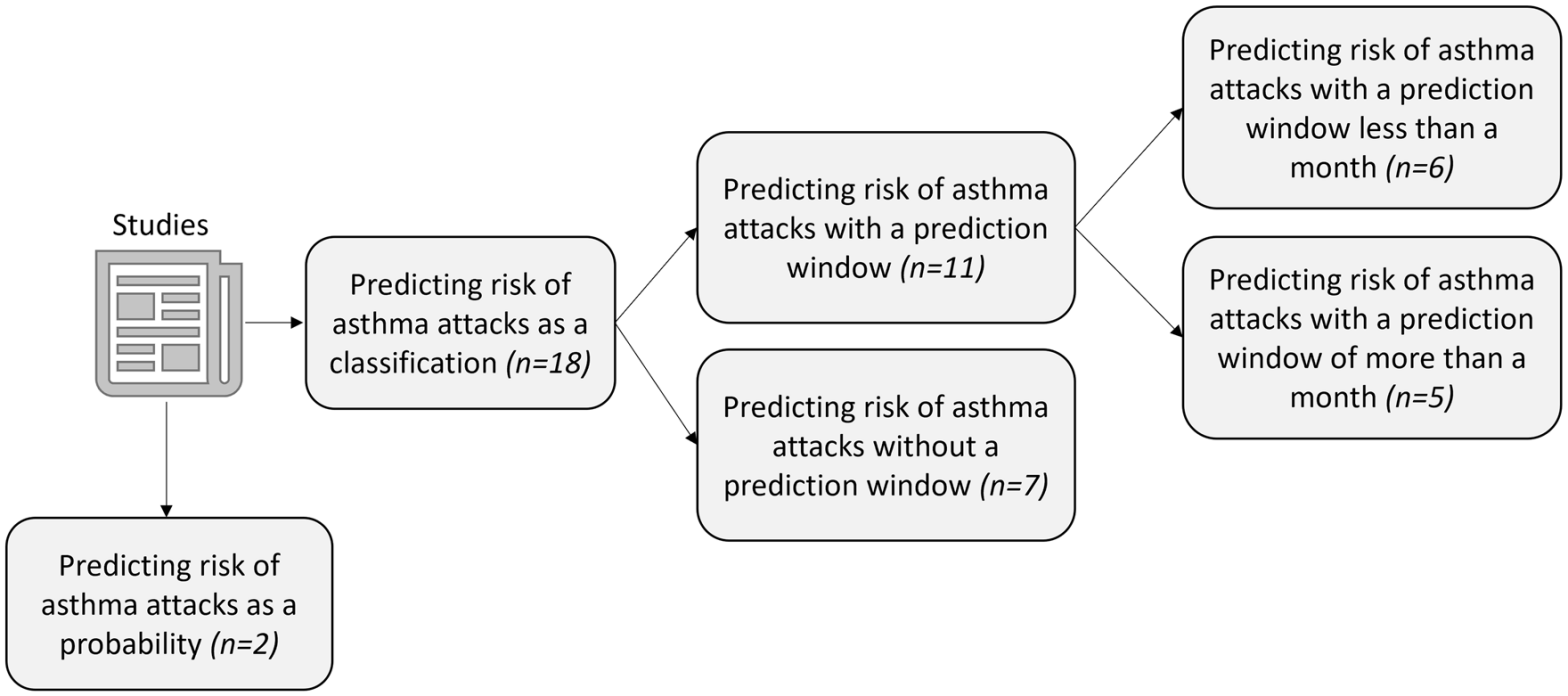
Presentation of the results of the review

The 20 studies used different ML techniques to predict the risk of asthma attacks. The outcome, asthma exacerbation, was considered either as a categorical variable or as a continuous variable in the form of a probability. Therefore, the studies can be categorised into 2 groups: 1) studies that predict the risk of asthma attacks as a classification (n=18) and 2) studies that predict the risk of asthma attacks as a probability (n=2). The classification group was further divided into 2 groups: studies with (n=11) and without (n=7) a prediction window. The studies with a prediction window can again be subdivided based on the window size: less than (n=6) and more than (n=5) a month. This categorisation of the studies is illustrated in Fig. 4.

In the literature, many studies predicted the risk of asthma exacerbations without considering the temporal effect. For instance, the impact of weather data from the previous day or a few days ago that might have triggered the symptoms of asthma patients. Therefore, it is critical to consider the impact of the different factors from previous days (lags) in forecasting the risk of asthma attacks. Further, instead of just making a prediction, a group of studies constructed models to predict asthma attacks for a specific time (prediction window), such as the coming 3 days, 7 days, 3 months, 1 year, and so on. Details about these models are presented in the following sections.

Among the ML algorithms employed in these studies, logistic regression (LR), decision trees (DT), random forest (RF), gradient boosting machines (GBM), extreme gradient boost (XGB), support vector machines (SVM), and neural network (NN) algorithms were used most often. Most of the studies exercised the *k-*fold cross-validation technique to validate the model on the training data. Different studies chose different *k* values such as 3 [38], 4 [27, 28], 5 [16, 23, 24, 32, 34] and 10 [30, 33, 35].

### Hyperparameter Tuning

Hyperparameters in ML models are external configuration settings that are not learned from the data but are set prior to the training process. They influence the overall behaviour of the model and affect its performance. Hyperparameters can be optimised using different techniques. Among the studies included in this review, only a very few [24, 34, 38, 40] conducted hyperparameter tuning. The grid search technique was applied in two of these studies [24, 34] while the randomised search technique was applied in another one [38]. (There are not enough details regarding the hyperparameter tuning process in [40].) Table 3 presents the details of the hyperparameter tuning conducted by past studies. It shows the various hyperparameters tuned with different values and techniques.

### Model Performance

The studies used different evaluation metrics to evaluate and compare the performance of the models, as shown in Table 4. These metrics were predominantly accuracy, area under the receiver operating curve (AUC-ROC), specificity, sensitivity, positive predictive value (PPV), and negative predictive value (NPV). Accuracy is the ratio of correctly predicted outcomes and the total number of samples, simply the overall correctness of the model. The AUC-ROC value represents the capability of the model to distinguish between the classes. Sensitivity, also called recall, measures the completeness of positive predictions, while specificity measures the completeness of negative predictions. PPV, also called precision, is the accuracy of positive predictions, while NPV is the accuracy of negative predictions.

**Table 3:** Hyperparameter optimisation details of the previous studies

**Study**	**Model**	**Hyperparameters**	**Values**	**Technique**
[34]	DT	tree depth	2, 4, 6, 8 ,10, 12	grid search
		split criterion	gini, entropy	
	GBM	maximum depth	3,7,9,11	grid search
		subsample	0.5, 0.7, 1	
	LR	solver	newton-cg, lbfgs, liblinear	grid search
		C	0.01, 0.1, 1, 10, 100	
	RF	subsample	10, 100, 1000	grid search
		maximum features	sqrt, log2	
	SVM	C	0.1, 1, 10, 100, 1000	grid search
		kernel	radial basis function, polynomial, sigmoid, linear	
[24]	XGB	number of trees	25, 100, 200	grid search
		maximum depth	1, 3, 5, 7, 8, 9	
	one class SVM	learning rate	0.1, 0.3, 0.5, 0.7, 0.9	grid search
		nu	0.001, 0.0015, 0.002, 0.004, 0.006, 0.008, 0.01	
		gamma	0.001, 0.01, 0.1, 1	
	LR	penalty	0.1, 0.2, 0.3, 0.4, 0.5, 0.6, 0.7, 0.8, 0.9	grid search

**Table 4:** Performance of the ML models developed for asthma risk prediction

Application type	Study	Best model (overall)	AUC-ROC^a^	AUPRC^b^	Accuracy	Specificity	Sensitivity	NPV^c^	PPV^d^
Classification - Without prediction window	[34]	GBM	0.97	-	97%	-	0.98	-	-
	[35]	NB	-	-	70.7%	0.73	0.70	0.53	0.85
	[36]	NN	-	-	94%	-	-	-	-
	[37]	BPNN	-	-	86%	0.86	0.85	-	-
	[38]	XGB	0.84	-	-	-	-	-	-
	[39]	RF	0.50	-	-	-	-	-	-
	[40]	XGB	Non-severe: 0.71	-	-	0.67	0.64	0.78	0.51
			ED visits: 0.88	-	-	0.76	0.84	0.99	0.12
			Hospitalisation: 0.85	-	-	0.73	0.86	1.0	0.05
Classification - With prediction window	[24]	LR	0.88	-	-	-	-	-	-
	[25]	CART	-	-	80.9%	0.97	0.65	-	-
	[26]	ABN	-	-	100%	1.00	1.00	-	-
	[29]	XGB	0.76	-	-	-	-	-	-
	[30]	Primary outcome: XGB	0.74	-	-	-	-		
		Secondary outcome: RF	0.68						
	[33]	Elastic-net LR	0.70	-	-	0.57	0.72	-	-
	[27]	Patients w/o comorbidities: XGB	0.90	0.007	-	-	0.007	-	0.03
		All patients: LightGBM	0.90	0.01	-	-	0.01	-	0.03
	[31]	XGB	0.86	-	-	-	-	-	-
	[28]	XGB	0.83	-	-	-	-	-	-
	[32]	RF	-	-	66.05%	0.68	0.65	-	-
	[23]	LR	0.83	-	-	0.83	0.90	-	-
Probability prediction	[41]	LSTM	RMSE^e^ = 0.093	-	-	-	-	-	-
	[16]	TSANN	0.69	-	-	-	-	-	-

^a^Area under the receiver operating characteristic curve

^b^Area under the precision-recall curve

^c^Negative predictive value

^d^Positive predictive value

^e^Root Mean squared error

### Predicting Risk of Asthma Attacks As a Classification

Nineteen of the previous studies predicted the risk of an asthma attack as a binary classification, and one study [37] considered asthma attacks as a multi-class classification problem. Most studies in the binary category predicted the presence or absence of an asthma attack, while others considered different levels of asthma attack, such as mild, moderate, or severe. This section discusses the studies that predicted the risk of asthma exacerbation as a category. Furthermore, imminent attacks could be predicted for a future time frame, for instance, the possibility of an attack in the next 3 days. While some studies constructed models by taking prediction windows into account, others did not. The following sub-section describes these groups.

#### Predicting Risk of Asthma Attacks With a Prediction Window of Less Than a Month

**Fig. 5:**
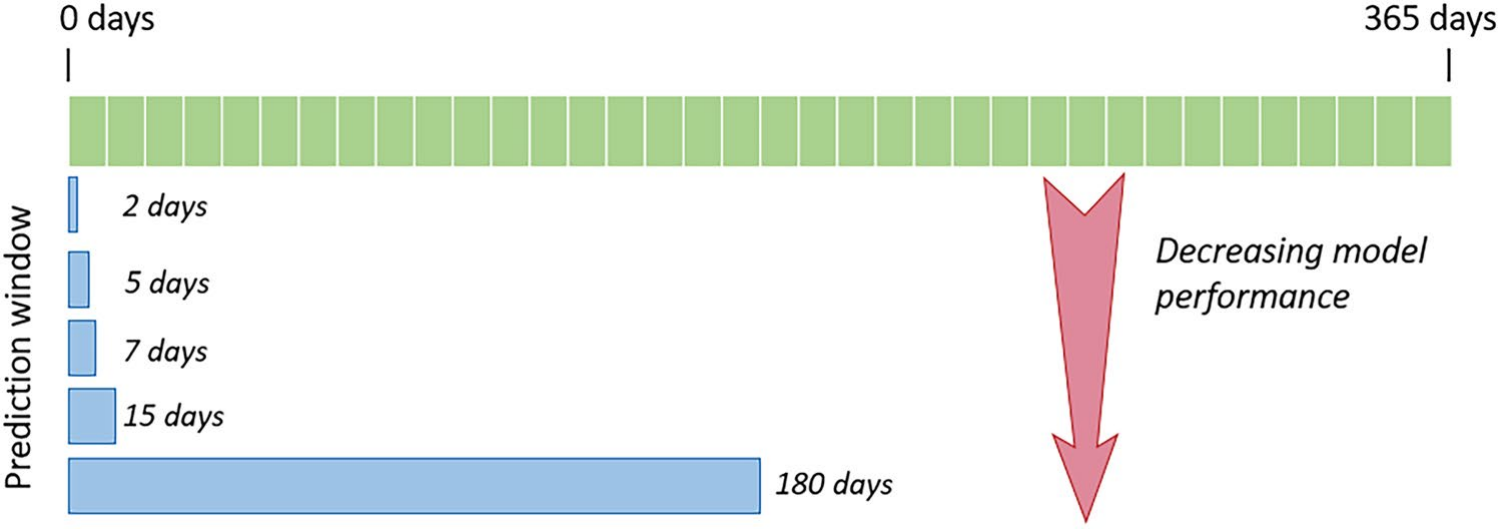
Association between prediction window size and model performance

In the literature, many studies predicted the risk of asthma exacerbations without considering the temporal effect. For instance, the impact of weather data from the previous day or a few days ago might have triggered the symptoms of asthma patients. Therefore, it is critical to consider the impact of the different factors from previous days (lags) in forecasting the risk of asthma attacks. Further, instead of just making a prediction, a group of studies constructed models to predict asthma attacks for a specific time (prediction window), such as the coming 3 days, 7 days, 3 months, 1 year and so on. Figure 5 depicts the association between prediction window size and the model’s performance. The figure highlights that the shorter the prediction window, the higher the model’s performance. The following section discusses those studies. We synthesised these studies into two categories according to the size of the prediction window as follows. This section represents the studies that classified the risk of asthma attacks using prediction windows for less than a one-month period. Table 5 in the Appendix shows a summary of the studies that developed ML models to predict asthma risk as a category using the prediction window concept.

Six studies [23–28] developed models for short-term prediction of severe asthma exacerbations. Five studies [23–26, 28] kept the prediction window at less than a week while one study [27] used more than 2 weeks (15 days) for the prediction window. In training the ML models, the authors used data from several previous days, which they defined as a lookback window. Four of the studies [24–27] applied the lookback window concept with the size of the lookback window ranging from 5 to 365 days for near-term prediction. However, [27] included inputs such as count of events for multiple lookback windows sizes - 10, 30, 60, 90, and 365 days. With the aim of exploring telemonitoring data for asthma risk prediction, and using the minimum description length (MDL) principle, [26] found that the telemonitoring alert (out of four zones) on day 7 has higher importance in predicting the asthma risk on day 8. Comorbidity burden and previous exacerbations were important predictors, identified through collinearity [27]. Even though they have implemented principal component analysis (PCA) and recursive feature elimination techniques to identify important features, those are not clearly stated in the article. Most works used tree-based algorithms such as DT [23], RF [27, 28], XGB [24, 27, 28], and CART [25]. Studies also developed models using LR [23, 24, 27, 28], SVM [24, 26], and NN [23, 27] algorithms. Only two studies applied data imbalance handling techniques- random under-sampling [23, 27], random over-sampling [23], and synthetic minority oversampling technique (SMOTE) [23]. There is no clear data available for data imbalance handling in other works.

#### Predicting Risk of Asthma Attacks with a Prediction Window of More Than a Month

A set of studies defined their prediction window size as greater than or equal to a one-month period. One study [29] used a 1-month period while [30] used 6-months period as the prediction window size. All of the other studies [31–33] kept the prediction window size to 1 year. While [29, 30, 32] considered lookback windows similar in size to prediction windows, no clear details of lookback windows are provided in [31, 33]. One study [29] identified clinical factors such as obesity, atopy, medication, asthma controller plan and patient service utilisation history as important asthma risk predictors. Asthma medication also played an important role in the research by [33]. Further, previous asthma exacerbations and length of treatment with biologics were key predictors of asthma risk in [30]. Meanwhile, age, hospital stay, blight prevalence, and neighbourhood inequality are important predictors, according to another study [32]. In developing prediction models, the most common ML algorithms utilised by these studies are LR [29, 30, 33], RF [29, 30, 32, 33], and XGB [29, 31, 33]. Only one study [32] in this category applied random undersampling to handle data imbalance.

#### Predicting Risk of Asthma Attacks Without a Prediction Window

This section focuses on the studies that developed ML models to predict the risk of asthma attacks without considering a prediction window. Table 6 represents the study summary for these studies. Seven studies [34–40] are included in this category and six of them considered this to be a binary classification problem. The seventh study [37] defined three target classes for risk as low, medium and high. A few studies specifically chose hospitalisations [38, 40] or ED visits [40], due to asthma attack being the target variable. Only two studies exercised feature selection. While [34] used the LASSO regression technique, [39] did feature selection with RF. With the aim of utilising environmental triggers and bio-signals for asthma risk prediction, one study [34] identified temperature, wind speed, relative humidity, air quality index (AQI), smoking status, allergies, normal PEFR and daily readings, asthma symptoms of the last 4 weeks, and number of asthma attacks for the last 4 weeks as important predictors. Incorporating other data with community viral load, [38] determined the key predictors as patient vital signs, acuity, age, weight, socio-economic status, dry bulb temperature, wind speed, and station pressure. Another work [40] mentioned the key features as age, long-acting beta-agonist, high dose inhaled glucocorticoid or chronic oral glucocorticoid therapy, FEV1, and FEV1 to FVC ratio. For developing the prediction models, commonly used ML algorithms were, DT [34, 35, 38], RF [34, 38–40], GBM variations [34, 38, 40], LR [34, 38, 40], and SVM [34, 35, 37]. Additionally, some authors experimented with Naive Bayes (NB) [35] and NN extensions [36, 37]. Only one of the studies [35] did not possess a high imbalance data set. However, all the other works had highly imbalanced data sets and they have not tried to handle it, other than [34], who applied SMOTE with SVM technique to handle data imbalance.

### Predicting Risk of Asthma Attacks as a Probability

Most of the previous studies on predicting the risk of asthma attacks addressed this risk as a binary or multi-class classification scenario. However, a prediction generated as a probability gives more meaning to the output. Rather than saying yes or no, a probability indicates the likelihood of something happening. Furthermore, a prediction presented as a risk score will help the medical practitioner better understand the status of the asthma patient. Accordingly, in the current review, we identified a few studies that used a probability or a score as the prediction outcome, which is clarified in this section. Table 7 summarises the studies that predict the risk of asthma attacks as a probability.

One study [41] presents a Deep Q-learning Network (DQN) framework to predict asthma attacks using the Q-learning method and LSTM to consider the personal risk scores of triggers. The authors calculated a relative risk score as the probability of an event in the exposed group divided by the probability of an event in the not-exposed group. The authors used this separately with vital signs and environmental time series data and they updated each record with a calculated personalised relative risk, presented as a numeric value. The deep learning algorithm, LSTM, was employed to predict the relative risk of asthma patients, which is then utilised in the Q-learning algorithm. Similarly, another study [16] was conducted to predict the risk of asthma exacerbations while exploring the potential risk factors using LSTM. They proposed a time-sensitive attentive neural network (TSANN) by applying an attention mechanism on both code-level and visit-level variables, which eased the model’s interpretability. They also added elapsed time embeddings into the model to indicate the relative time interval between each visit date and the prediction date. They identified gender, race, a few diagnoses and medications as important predictors of asthma attacks [16].

## Discussion

### Data for Predicting Risk of Asthma Attacks

**Fig. 6:**
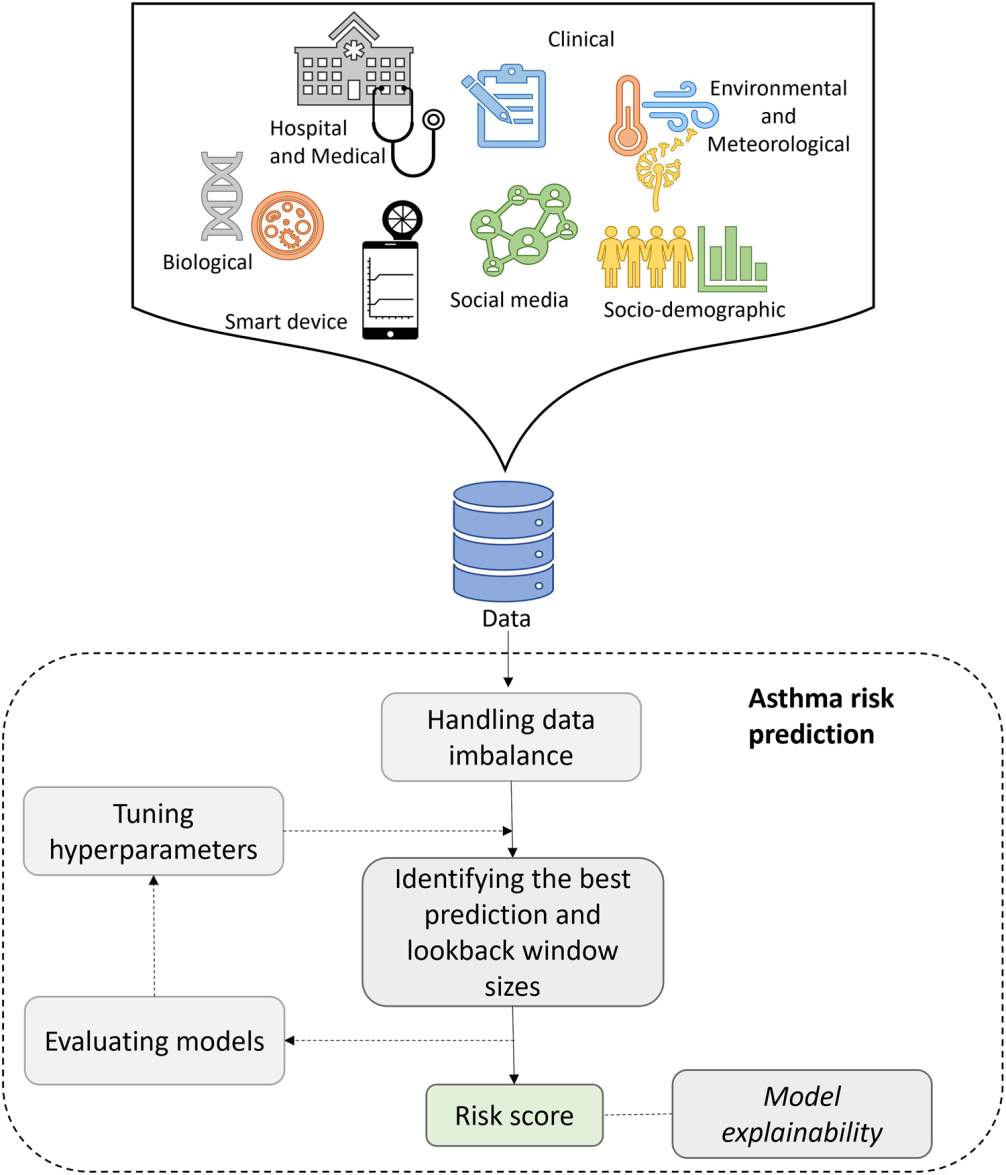
Conceptual overview for asthma risk prediction using ML

A conceptual model for predicting the risk of asthma attacks using machine learning techniques is presented in Fig. 6. As asthma is a health condition affected by many factors, data from heterogeneous domains have been integrated to predict the risk of asthma exacerbations listed in Table 2. Asthma is affected by socio-demographic factors, including age, sex, education level, socio-economic status, and marital status [42]. Socio-demographic features including age, gender, race, insurance type, and area of residence [16, 29, 30, 32, 33, 36, 39, 40] have been used with clinical factors such as asthma symptoms, PEFR, reliever medications, and inhalations [24, 25, 28, 34, 40]. Diagnoses, BMI, smoking status, previous asthma attacks, dispensed medications, Charlson Comorbidity Index, comorbidities, ED visits, and hospital visits [27, 29, 30, 33–35, 41] were embedded as hospital and medical factors.

Environmental and meteorological data can be used for health forecasting with respect to respiratory diseases [43]. Meteorological data, such as temperature, humidity, air pressure, and wind speed have impacts on asthma [7, 8]. Air pollutants can have chronic effects on humans and thereby pose a greater risk to a larger population. These factors are useful for predicting asthma since they could lead to a high number of asthma-related hospital admissions. Asthma triggers including temperature, relative humidity, wind speed, AQI, air pressure, PM_2.5_, pollen and gasses like CO, $NO_2$, $SO_2$ were the environmental and meteorological factors investigated in the predictor list by [29, 34, 36–38, 41]. Additionally, we found one study using the biological factor SNP [39].

Among several factors collected from multiple domains, previous studies have found that smoking status [34], normal PEFR and daily readings, previous asthma attacks, asthma symptoms, medication usage [16, 27–29, 33, 34], comorbidities [27–29], and hospital stays [32] are important predictors of future asthma attacks. Socio-economic factors, including SES [38], age [32, 38, 40], gender, and race [16], have shown to be important in predicting asthma attacks. Weather changes highly affect the control level of asthma patients, and accordingly, past studies show that temperature, wind speed, relative humidity,[34, 38], and AQI [34] have strong predictive power.

These results show the importance of including multiple factors from different domains (see Fig. 6). However, only a few studies have utilised predictors from more than three of the above heterogeneous domains. It seems important to incorporate different factors from multiple domains to forecast impending asthma attacks, as previous works have identified important predictors from each domain. Further, with the vast development of technology, smart devices such as smart peak flow meters and smart inhalers are in the hands of asthma patients. Utilising data from these devices will be more accurate than using data from daily diaries because manually entered data is prone to human errors and recall bias. Finally, social media usage has become a popular place where participants share their feelings and opinions. Therefore, it is worth investigating the use of social media data streams in asthma risk prediction.

### Handling Data Imbalance

In real-world data, especially health data, the existence of positive cases is very low compared to the negatives. For instance, the proportion of asthma patients who are admitted to hospitals due to asthma attacks is very low compared to the number of patients who are not admitted to hospitals. This kind of research aims to identify the minority class over the majority. Hence, class imbalance needs to be carefully addressed when applying ML models that highly depend on data. Otherwise, the model will not be able to learn the patterns of positive cases and thereby produce a lower performance for the minority class.

There are several ways to handle data imbalance. The most commonly used techniques are random under-sampling [23, 27, 32] and random over-sampling [23]. In random under-sampling, the number of records in the majority class will be reduced to match with the minority class, and it is vice versa for random over-sampling. Additionally, SMOTE is a more advanced technique that is used to handle data imbalance. An alternative to the random over-sampling technique mentioned above, SMOTE uses a more robust approach to generate new points based on the existing data. Further, the edited nearest neighbours (ENN) and Tomek link are under-sampling techniques that can be used for down-sampling data.

Data imbalance should be handled; otherwise, classifiers would be biased or generate misleading results [7, 27, 44]. However, applying a data balancing technique is not necessary, provided that the data set has considerable balance among target values. It should be noted that these data balancing techniques need to be applied only on the training set; otherwise, a data leakage issue arises. These techniques may improve the predictions in some cases by artificially eliminating or simplifying the records in the data set. The prediction model using the random under-sampling technique showed the best performance with higher specificity and sensitivity values compared to the other models (see Table 3). This highlights the importance of addressing data imbalance prior to developing the ML models.

### ML Techniques in Asthma Risk Prediction

Clinical practices are mostly dependent on the professional skills that individuals acquire with practice and experience. However, the human brain has limited abilities to identify longitudinal patterns from massive amounts of data [14]. ML-based models can assist in analysing the growing amount of patient data, achieving patient-oriented decisions [45]. ML techniques can discover undisclosed patterns and find new information from the data. Costs incurred in healthcare could be reduced with the support of ML. Even though the knowledge and experience of a physician are irreplaceable, computer-aided support could increase the efficiency and accuracy of the diagnostic process. Therefore, ML could play a significant role in diagnosing and predicting asthma.

There are several machine learning algorithms that previous studies have used to predict imminent asthma attacks, as mentioned in Tables 5 and 6. In terms of classifying a patient to have or not have an asthma attack in the future, XGB, RF, and LR algorithms have achieved the best performance compared to other classifiers, regardless of the presence of a prediction window or not (see Table 4). On the other hand, both studies that predict the risk probability have used extensions of NN. In contrast to predicting the risk of asthma attacks as a category (yes or no), the probability of having an asthma attack will deliver more information to the health practitioner. The limited number of studies in this review that utilised probability prediction (see Table 7) emphasise that further research is required to understand the applicability of ML techniques in predicting the risk of asthma attacks as a probability.

### Prediction and Lookback Window

When anticipating the risk of attacks, it is more useful if the prediction can be made within a specified time window. As presented in the “Results” section, researchers set the prediction window from 1 day [25, 26] to 1 year [31–33]. However, none of the studies mentioned a specific reason for choosing the window size. They used either a random value or chose the window size according to the nature of the dataset. From the analysis of the results of those models, we identified that short-term predictions achieved better performance compared to long-term predictions (refer to Fig. 5). In parallel, the researchers fed the past data into the prediction models within a lookback window, which is also called lag (i.e. past data for a given period of time). Incorporating historical data is crucial, especially when considering more common triggers, such as weather factors. While most of the studies have considered lookback windows, there is no clear information in defining the window size. Also, it seems that they have defined the window size according to the availability of data. There is a lack of knowledge about the optimum size of the lookback and prediction windows in predicting the risk of asthma attacks. Hence, it is important to explore and determine these windows for an accurate prediction.

### Generating an Explainable Risk Score

Different clinical tools have been developed for monitoring and managing asthma. Global Initiative for Asthma (GINA) guidelines have been formulated based on the level of symptoms in the last four weeks to differentiate between controlled, partially controlled, and poorly controlled asthma. The Asthma Control Test (ACT) [46] and Asthma Control Questionnaire (ACQ) are the most common clinical scores used for asthma control. A study [47] predicted the Pediatric Asthma Risk Score (PARS) with a logistic regression model by assigning weights to the predictors using the rounded odds ratios. Many studies [48–50] have used multivariate logistic regression to develop a risk score. Accordingly, past models have considered a linear relationship between the predictors in generating the risk score or used the manually calculated scores as the target outcome of ML models. However, when the number of features becomes high, it is unreasonable to assume that they always have linear relationships. Therefore, developing a new asthma risk score requires more advanced technologies, such as machine learning, which can handle non-linearity among the predictors. While using these techniques, the prediction should be explainable to the clinicians in order for them to trust the score. This transparency is essential in the healthcare industry. The existing asthma risk scores are derived mainly from demographic and clinical factors. Instead of considering only the subjective factors, such as the prevalence of symptoms for a period, it is important to examine objective factors, such as peak flow and inhaler data, in deriving a control score. Asthma is a multi-factorial disease; it triggers patients’ disease levels based on individual factors. Therefore, a more objective continuous score is necessary to predict the asthma control level. Further, rather than keeping the model a “black box”, adding the reason behind the generated value would add more value and transparency to the prediction. It adds interpretability to the model which will be clinically important.

### Hyperparameter Tuning

Machine learning algorithms comprise diverse parameters assigned varying values to optimise performance. Among these, model parameters, internal in nature, undergo configuration during the learning process as the model adapts to data provided to it. A prime example is the weights of a neural network, acquired during the training phase. Conversely, hyperparameters, external in nature, necessitate the assignment of values before the training stage. Identification of hyperparameter values, termed hyperparameter optimisation or tuning, precedes model training. For instance, determining the optimal learning rate for a neural network is pivotal for achieving peak model performance. In practice, iterative fine-tuning of hyperparameters involves training multiple models with diverse parameter combinations, evaluating performance, and selecting the most optimal model. This procedure can be executed by utilising the default values for the algorithm, manually configuring them based on past examples or experiments, or seeking assistance from experts to define these parameters [51]. Random searching [52] is one of the strategies for hyperparameter tuning that will consider a pool of values and choose different combinations randomly to find the best set of hyperparameter values. On the other hand, in the grid search technique, all the combinations of the specified set of values will be tried to determine the best combination which gives the highest model performance. A study compared the grid search and random search techniques to predict chronic kidney failure using XGB [53]. The best results came from the grid search technique. There are other hyperparameter optimisation techniques such as Bayesian optimisation [54, 55], genetic algorithm, etc. Experimental outcomes were comparable; nevertheless, the genetic algorithm outperformed the grid search technique and the Bayesian algorithm to predict customer transactions using NN [56]. However, except for a few works, the studies we reviewed lacked this important step. The ultimate goal of constructing these ML models for risk prediction is to gain the highest performance for the given problem. Therefore, conducting hyperparameter optimisation is crucial in developing ML models as it can improve the models’ performance. Researchers must delve into suitable techniques for tuning hyperparameters in the machine learning models they construct.

## Conclusion

With growing ML applications in healthcare, several studies have employed these advanced computer-based techniques for predicting impending asthma attacks using heterogeneous data sources. In this review, firstly, we have presented a conceptual overview of the landscape of this research so that researchers can engage in future research in a similar context (see Fig. 4). Secondly, this review has generated a list of data sources available for researchers, which is available in Table 2. Thirdly, for each branch of the review, we presented lists of studies predicting the risk of an asthma attack as a classification with and without a prediction window (Tables 5 and 6) and prediction as a probability (Table 7).

The findings of this review confirm the importance of using different predictors from multiple data sources for predicting asthma attacks. The analysis shows the applicability of ML algorithms and their ability to perform on asthma patient data, including other factors. For classifying future attacks, ensemble methods based on decision trees have given better performance, while extended neural networks have achieved acceptable results on probability prediction. Further, the summary of the past studies confirmed that having a shorter prediction window improves the prediction outcome. For future research in ML applications in asthma prediction, hyperparameter tuning and data imbalance handling need to be carefully followed to integrate data from multiple sources. Additionally, optimal lookback and prediction windows for asthma risk prediction require further investigation. Moreover, deriving an explainable asthma risk score with the integration of multiple data sources using ML techniques needs to be explored which will assist health practitioners.

## Supplementary Information

Below is the link to the electronic supplementary material.

Supplementary file 1 (pdf 415 KB)

## Data Availability

Not applicable.

## Appendix

**Table 5:** Summary of the studies on predicting risk of asthma attacks as a classification - with prediction window

Study	Study aim	Important predictors identified	Feature selection	Data balancing	Target	ML algorithms	Prediction window	Lookback window	Outcomes/Findings
[24]	Develop and validate prediction models for short-term prediction of severe asthma exacerbations	Not given	None	None	Occurrence of a severe asthma exacerbation(0 or 1)	XGB, SVM, LR	2 days (same day or next day)	5 days	ML models reached higher discriminative performance than a simple clinical rule
[25]	Develop an algorithm that predicts asthma exacerbation one day in advance based on the previous 7-day window	Not given	Not given	Not given	Asthma exacerbation ("no–alert" or "high–alert")	CART	1 day	7 days	Developed a model that predicts asthma exacerbation on next day based on the previous 7-day window
[26]	Explore the use of telemonitoring data for building ML algorithms that predict asthma exacerbations	Zone 7	MDL	Not clear	Asthma exacerbation ("no-alert" or "high-alert")	NB, ABN, SVM	1 day	7 days	Demonstrated the potential of ML techniques in personalised decision support, found the attributes of day seven alone had more predictive power
[29]	Develop a clinical decision support tool to predict the risk of asthma exacerbation in children on a monthly- basis	obesity, atopy, medication, asthma controller plan, patient service utilisation history	None	None	Asthma exacerbation (0 or 1)	LASSO, RF, XGB	30 days	30 days	Near-term prediction models performed better, and clinical factors alone had more predictive power
[30]	Develop a model to predict asthma exacerbation in the 6 months after stopping biologic therapy using ML techniques	previous asthma exacerbations, length of treatment with biologics	None	None	Asthma exacerbation (0 or 1)	Elastic-net LR, RF, GBM	6 months	6 months	ML algorithms performed moderately in predicting asthma attacks after stopping asthma biologics
[31]	Develop a method which automatically explains the results of an asthma risk prediction model on imbalanced tabular data	Not given	None	None	Asthma hospital visit (0 or 1)	XGB	1 year	Not given	Asthma risk prediction model with automatic explanation component
[27]	Develop ML models for predicting risk of asthma exacerbations	comorbidity burden, previous exacerbations	Collinearity	Random Under-sampling and Weighting	Asthma exacerbation (moderate or severe)	RF, XGB, RNN, LR(LASSO, Ridge, Elastic-net)	15 days	Multiple (10, 30, 60, 90, 365 days)	Prior clinical information is not sufficient for near-term asthma risk prediction
[28]	Discover the usability of digital inhaler data merged with demographic and clinical data for predicting future asthma exacerbations	mean number of daily inhalations during prior 4 days, comparison to baseline inhalation parameters, previous exacerbations and comorbidities	None	None	Asthma exacerbation (0 or 1)	LR, RF, XGB	5 days	Not given	Digital inhaler data with demographic and clinical factors can be used for predicting impending asthma exacerbations
[32]	Analyse the impact of using demographic, social, and symptoms related features in asthma hospital re-visit prediction on pediatric asthma patients	age, hospital stay (duration), blight, neighbourhood inequality	None	Under-sampling	A second hospital visit (0 or 1)	RF, SVM	1 year	1 year	Socio-markers can be reliable predictors of hospital re-visits and combined demographics, socio-markers and biomarkers can predict future hospital re-visits moderate accurately
[33]	Predict asthma exacerbations in the next year with the use of electronic data conditional on previous adherence to asthma medications	Asthma medications	None	None	Asthma exacerbation (0 or 1)	Elastic-net LR, RF, XGB	1 year	Not given	Inclusion of medication-related covariates increase the odds of having as asthma exacerbation
[23]	Derive and validate a model predicting future exacerbations using telemonitoring data	Not given	PCA, Recursive Feature Elimination	Random over-sampling, Random under-sampling, SMOTE	Asthma exacerbation (0 or 1)	LR, NB, DT, NN	4 days (same day or next 3 days)	None	ML models combined with telemonitoring data such as PEFR

**Table 6:** Summary of the studies on predicting risk of asthma attacks as a classification - without prediction window

Study	Study aim	Important predictors identified	Feature selection technique	Data imbalance handling	Target	ML algorithms	Outcomes/Findings
[34]	Utilising bio-signals and environmental triggers for asthma risk prediction	smoking status, allergies, normal PEFR and reading, asthma symptoms (last 4 weeks), medication (last 4 weeks), no. of asthma attacks (last 4 weeks), temperature, wind speed, relative humidity, AQI	LASSO Regression	SMOTE using SVM (SMOTESVM)	Asthma attack (0 or 1)	DT, GBM, LR, RF, SVM	DT has the poorest performance while GBM has the best performance
[35]	To evaluate models for predicting the severity of asthma exacerbation in ED visits, constructed from data using ML techniques and to select the best-performing model to compare with Pediatric Respiratory Assessment Measure (PRAM) score and predictions made by ED physicians	Not identified	Not conducted	N/A (data not highly imbalanced)	Asthma exacerbation (Mild or Moderate/Severe)	NB, DT, E-DT, SVM, IB1, IB10	NB performed better among ML models, but both the PRAM score and the NB model were less accurate than physicians
[36]	Exploring the impact of weather on asthma exacerbations and examines the effectiveness and limitations of several recent asthma self-management tools and applications	Not identified	Not conducted	None	Asthma attack(low or high)	NN	Personalisation has positive effects on asthma self-management, and user demographics and weather have positive effects on personalised weather-based healthcare
[37]	Determine the severity of asthma using real-time data from medical IoT devices while monitoring continuously	Not identified	Not conducted	None	Risk of asthma (low, medium, high)	BPNN, SVM, KNN	BPNN achieved higher accuracy than other classifiers
[38]	Compare the performance of ML approaches incorporating a few data domains with community viral load for early prediction of the need for hospital-level care in pediatric asthma	patient vital signs, acuity, age, weight, socioeconomic status, dry bulb temperature, relative humidity, wind speed, and station pressure	None	None	Hospitalisation (0 or 1)	DT, LASSO Regression, RF, XGB	Except DT, all other models perform well in prediction and adding weight, SES, and weather data improved the model performance
[39]	Validating the hypothesis that the use of RF classifier to select SNPs would result in an improved predictive model of asthma exacerbations	set of SNPs	RF	None	Severe asthma exacerbation (0 or 1)	RF	RF modelling can produce accurate results using hundreds of SNPs and RF-selected SNPs contain information about exacerbation, while the random SNPs do not
[40]	Develop ML models with large-scale and real-world local data to predict asthma exacerbations	age, long-acting $\beta$ agonist, high-dose inhaled glucocorticoid, or chronic oral glucocorticoid therapy, low FEV1 and FEV1 to FVC ratio	None	None	None-severe exacerbation (0 or 1); ED visits (0 or 1); Hospitalisation (0 or 1)	LR, RF, LightGBM	Higher albumin was related to lower risk of asthma exacerbation, and for patients with low FEV1 or FEV1 to FVC ratio, these features significantly affect their individual risk prediction

**Table 7:** Summary of the studies on predicting risk of asthma attacks as a probability

Study	Study aim	Important predictors	Feature selection technique/s	Data balancing	Target	ML algorithm	Outcomes/Findings
[41]	Develop an asthma risk prediction model by investigating the ability of Q-learning method	None	None	None	Relative risk (numeric)	LSTM	DQN framework for predicting asthma risk with the use of personal risk scores of triggers
[16]	Predict the risk of asthma exacerbations and explore potential risk factors	Gender, race, some diagnosis codes and medications	Attention weights of the model	None	Asthma exacerbation (probability)	TSANN	A TSANN model employing self-attention on both code-level and visit-level and addition of elapsed time improved model performance
