# Supplementary material for: Investigating Machine Learning Techniques for Predicting Risk of Asthma Exacerbations: A Systematic Review
Source: J Med Syst. 2024 May 13;48(1):49. doi: 10.1007/s10916-024-02061-3 (PMC11090925; doi:10.1007/s10916-024-02061-3)
Supplement: Supplementary file 1 — (pdf 415 KB) [file 10916_2024_2061_MOESM1_ESM.pdf]

Table S1: Results of risk of bias assessment

Author & year	Is the rule defined adequately	Representativeness of the cases	Selection of controls	Is the methods and techniques used to construct and validate the models clearly explained	Is the model reliable
Alharbi 2022	✓	✗	✗	✓	✓
deHond 2022	✓	✓	✓	✓	✓
doQ 2019	✓	✓	✗	✓	✓
Farion 2013	✓	✓	✓	✓	✓
Finkelstein 2016	✓	!	✗	✗	!
Finkelstein 2017	✓	✓	✗	✓	✓
Haque 2021	✓	✗	✗	✓	!
Hurst 2022	✓	✓	✓	✓	✓
Inselman 2023	✓	✓	✓	✓	✓
Jiao 2022	✓	✓	✓	✓	✓
Lisspers 2021	✓	✓	✗	✓	✓
Lugogo 2022	✓	✓	✓	✓	✓
Luo 2020	✓	!	✗	✓	✓
Patel 2018	✓	✓	✗	✓	✓
Priya 2021	✓	✓	✗	✓	✓
Shin 2018	✓	✓	✓	✓	!
Xiang 2019	!	✓	✓	!	!
Xu 2011	✓	!	✓	!	!
Zein 2021	✓	✓	✓	✓	✓
Zhang 2021	✓	✓	✗	✓	✓

Hgh quality	
Some concerns	
Low quality	

**Is the rule defined adequately:** Has they define the prediction rule clearly
**Representativeness of the cases:** Selection of an appropriate spectrum of patients to train the models
**Selection of controls:** Was the models validated in a different group of patients
**Is the methods and techniques used to construct and validate the models clearly explained**
**Is the model reliable:** Does model perform well
